# Melting Flow in Wire Coating of a Third Grade Fluid over a Die Using Reynolds’ and Vogel’s Models with Non-Linear Thermal Radiation and Joule Heating

**DOI:** 10.3390/ma12193074

**Published:** 2019-09-20

**Authors:** Zeeshan Khan, Waqar A. Khan, Haroon Ur Rasheed, Ilyas Khan, Kottakkaran Sooppy Nisar

**Affiliations:** 1 Sarhad University of Science and Information Technology Peshawar, KPK, Peshawar 25000, Pakistan; zeeshansuit@gmail.com (Z.K.); haroon.csit@suit.edu.pk (H.U.R.); 2 Department of Mechanical Engineering, College of Engineering, Prince Mohammad Bin Fahd University, Al Khobar 31952, Saudi Arabia; wkhan@pmu.edu.sa; 3 Faculty of Mathematics and Statistics, Ton Duc Thang University, Ho Chi Minh City 72915, Vietnam; 4 Department of Mathematics, College of Arts and Sciences, Wadi Al-Dawaser, Prince Sattam bin Abdulaziz University, Wadi ad-Dawasir 11991, Saudi Arabia; ksnisar1@gmail.com

**Keywords:** wire coating, third-grade fluid, heat transfer, non-linear thermal radiation, joule heating, pressure-type die

## Abstract

Wire coatings are necessary to provide protection from the aggressive environment and to add mechanical strength to wires and cables. In this study, we investigated the effect of radiative linear as well as non-linear heat transfer on the wire coating in response to joule heating, using a third grade fluid as the coating material. For the temperature dependent viscosity, two models namely—Reynolds’ and Vogel’s—were used. The non-linear ordinary differential equations were solved analytically by the Homotropy Analysis Method (HAM). Numerical technique was also applied for comparison and good agreement was found. It is interesting to note that the temperature parameter had a remarkable effect on the temperature distribution and heat transfer characteristics in the flow region within the die. It was observed that the velocity of the fluid within the die decreased as the magnetic parameter increased, while the magnetic field had an accelerating effect on the temperature distribution. Near the surface of the wire, the velocity of the coating material accelerated as the temperature parameter and radiation parameter increased. Analysis also showed that the temperature of the coating material decreased with increasing radiation and temperature parameters.

## 1. Introduction

Wire coating is an extrusion process commonly used in the polymer industry for the insulation of wires and cables. In this process, either a bare preheated wire is dragged through the extruded melted polymer or the melted polymer is extruded continuously over an axially moving wire. There are five units in a typical wire coating apparatus namely, a pay-off device, a wire preheater, an extruder equipped with an across-head die, a cooling trough, and a takeoff device. There are two kinds of cross-sectional dies that are commonly used in the wire coating analysis—the tubing-type die and the pressure-type die. The latter type of die is commonly used for wire coating. The pressure-type die closely resembles an annulus and therefore flow through this type of die has an analogy with the flow through the annular region formed by the two coaxial cylinders, out of which the inner cylinder is moving in the axial direction while the outer cylinder is fixed.

Studies on the wire coating for Newtonain as well as non-Newtonain fluids in a pressure-type coating die were carried out by pioneer researchers including Bernhardt [1], McKelvey [2], Bagley and Storey [3], Carleyeet et al. [4], and Han [5], who used the power-law and Newtonian models to describe the rheology of the melted polymers. The textbooks of Middleman [6] and Zeeshan et al. [7] also presented the analysis of the wire coating for pressure-type dies using the Newtonian and power-law fluid models. Later studies on this subject were carried out by Kasajima and Ito [8], Tadmor and Bird [9], Zeeshan et al. [10], and Wagner and Mitsoulis [11]. A detailed review of the literature on the flow of fluid and heat transfer in wire coatings up to 1986 was given by Mitsoulis [12]. A theoretical model to predict the pressure distribution within a stepped parallel bore wire coating unit was developed by Akter and Hashmi [13]. In a later study, Akter and Hashmi [14] simulated the polymer flow during the wire coating using a conical unit. Akter and Hashim [15] also presented the comparisons of experimental and theoretical results based on a non-Newtonian plastic–hydrodynamic model for wire drawing in a parallel and tapered bore unit.

**Figure 1:**
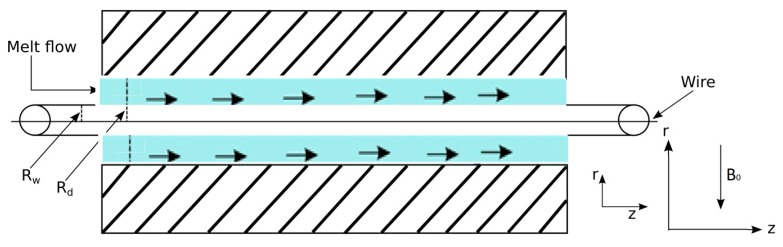
Geometry of the wire coating analysis.

One of the best qualities of non-Newtonian fluid is its viscoelastic property. Caswell and Tanner [16], Tucker [17], and Basu [18] described an operation in which either the polymer was extruded on an axially moving wire or the wire was dragged inside a die filled with the molten polymer. In this process of coating, the continuum of velocity and the melted polymer develops a high pressure in a specific region, which in turn produces a strong bond and imparts fast coating. The experimental set-up of a typical wire coating process is shown in Figure 1. In this set-up, the uncoated wire unwinds at the payoff reel and passes through a straightener, a preheater, and a crossshead die. Then the wire meets the melted polymer, emerges from the extruder, and gets coated. This coated wire then passes through a cooling trough, a capstan, and a tester to finally end on the rotating take-up reel. The co-extrusion process is simple to apply, time efficient and economical in the view of industrial applications.

Many researchers including Tadmor and Gagos [19], Mitsoulis [20], and Roy and Dutt [21] have contributed to this field of study. Siddiquiiet et al. [22] also analyzed wire coating using third-grade fluid and fourth-grade fluid. The third-grade fluid considered here represents a viscoelastic fluid of industrial importance. Many fluids used in wire coating exhibit the characteristics of third-grade fluid. Recently, a viscoelastic fluid model known as the Phan-Thien–Tanner (PTT) model is widely used for wire coating. Many authors have contributed to enrich the field of heat transfer in the post-treatment analysis of wire coating. Winter [23] extended the thermal analysis inside as well as outside the die. Symmons et al. [24] have studied plasto-hydrodynamic die-less wire drawing. Fenner and Williams [25] carried out an analysis of the flow in the tapering section of a pressure-type die. They obtained numerical solutions for the pressure and velocity profiles in the die. Further explanations were provided by Fenner [26], and these types of analyses were employed by the wire coating industry for tapered pressure-type dies. The properties of the final product greatly depended on the rate of cooling in the manufacturing processes. The central cooling system is beneficial to facilitate the process for a designed product. An electrically conducting polymeric liquid seemed to be a good candidate for some industrial applications such as in polymer technology and extrusion processes because the flow could be regulated by external means through a magnetic field. Applying a magnetic field may play an important role in controlling the flow momentum and heat transfer in the boundary layer of different fluids in the process of wire coating. In view of this, many authors have explored the effect of a transverse magnetic field on Newtonian and non-Newtonian fluids. Liu [27] considered the effect of subjecting an electrically conducting second-grade fluid to a transverse magnetic field past a stretching sheet. Salem [28] used a shooting technique to numerically study the effects of variable viscosities and thermal conductivities on the MHD flow and heat transfer of a viscoelastic fluid over a stretching sheet with a variable temperature. Shaheet et al. [29] have considered a third-grade fluid as a coating material in wire coating analyses in the absence of a magnetic field and used the perturbation method for obtaining an analytical solution. Further, Aksoyaand Pakdemirli [30], Siddiquieet et al. [31], and Siddiquieet et al. [32] have used a third-grade fluid in their study. Mishra [33] has considered the flow of a viscoelastic liquid in a circular cylinder. 

Shadloo et al. [34] used a viscoelastic fluid in the presence of a magnetohydrodynamic flow in the converging and diverging channel. A series simulation was obtained by applying the homotopy perturbation method. Maleki et al. [35] studied the heat transfer of non-Newtonian nanofluids imbedded in a porous medium. Shadloo et al. [36] studied the heat transfer of the series solution over a stretching sheet using the Homotropy Analysis Method (HAM). Zeeshan et al. [37] studied the effect of thermal radiation on non-Newtonain fluid through a porous medium and the analytical solution was obtained using HAM. Zeeshan et al. [38] obtained a numerical simulation using the Oldroyd 8-constant fluid as a coating material for wires. Mabood et al. [39] analyzed the magnetohydrodynamic boundary layer flow and heat transfer of nanofluids and a numerical simulation was obtained. Anuar et al. [40] investigated the flow of the boundary layer along with the slip condition over a moving plate of carbon nanotubes. Maleki et al. [41] observed the heat transfer and viscous dissipation of the pseudo-plastic nanofluid over an embedded porous plate.

Nayak et al. [42] explored the influence of a transverse magnetic field on the wire coating using a third-grade fluid as the coating material. This is one of the major 20th century contributions, regarding the flow as well as the heat transfer of a third-grade fluid on wire coating, to the development of a better-quality final product (coated wire), due to the better controlled rate of cooling. However, they did not investigate the influence of the linear as well as non-linear thermal radiation in their study.

The objective of the present study is to analyze the influence of linear as well as non-linear thermal radiation in the wire coating process, wherein a coating material is modeled as a third-grade fluid (non-Newtonian fluid) viz. melted polymer, and includes the temperature dependent viscosity in response to the Reynolds’ and Vogel’s models. The modeled non-linear equations were solved using HAM [34,35,36,37]. The effect of emerging parameters of Reynolds’ and Vogel’s models on the velocity and temperature profiles has been discussed through graphs. For the sake of validity and accuracy, the problem was also solved by applying a numerical technique [38,40] and a comparison was done with the published work [43]. 

## 2. Formulation of the Problem 

Consider the boundary layerrflow of an incompressible third-grade fluid such as a molten polymer like polyvinyl chloride (PVC), inside a stationary pressure-type die of finite length *L* having radius *R_d_* and temperature $\Theta_{d}$. Suppose a wire of radius *R_w_* is extruded along the axis of the die with velocity *U_w_* and temperature $\Theta_{R}$ as shown in Figure 1. Let us make the following assumptions—(1) the flow is steady; (2) the melted polymer flows through a suitably long cylindrical die in which a wire moves axially at a constant speed; (3) the flow is laminar; (4) the velocity in the radial direction is negligibly small compared to that in the axial direction; (5) the inertial effect is negligibly small compared to viscous effect that is reasonably large due to the extremely high viscosity of the melted polymer; (7) the excessive wall shear stress is avoided as it may lead to elongation or frequent breakage of the wire in the coating operation, and may also cause uneven and rough extruded coating; (8) heat conduction in the direction of flow is negligibly small compared to that in the radial direction; (9) the melted density, specific heat, and thermal conductivity are independent of temperature, while the viscosity depends on temperature; (10) the no-slip boundary conditions are subjected to the moving wire as well as the stationary die wall; (11) the gravitational effect is negligible; and (12) the fluid is acted upon by a constant pressure gradient $\frac{dp}{dz}$ in the axial direction. 

The wire and die are concentric and a cylindrical co-ordinate system (r,z) was chosen at the center of the wire to analyze the flow situation where z- and r-axes were taken along and perpendicular to the direction of flow respectively. The design of the wire-coating die was of primary importance since it significantly affected the quality of the final product. The pressure-type die was considered because within this die, the melted polymer met the wire at a location where a complex flow field existed, and its understanding was vital for the better design of dies with optimum performance. Considering the above-mentioned frame of reference and assumptions, the fluid velocity, extra stress tensor, and temperature field was defined as:

(1) V=[0,0,w(r)], S=S(r) and Θ=Θ(r).

The equations of the continuity, momentum, and energy governing the flow of an incompressible fluid are:

(2) ∇·V=0,

(3) $$\rho \frac{DV}{Dt}=-\nabla p+\nabla \cdot S+J\times B,$$

(4) $$\rho C_{p}\frac{D\Theta }{Dt}=k\nabla^{2}\Theta +\phi -q_{r}^{\prime }+J_{h},$$

where ∇⋅S is the viscous force, ϕ=S:∇V is the viscous dissipation, $q_{r}^{\prime }$ is the radiative heat flux so that $q_{r}^{\prime }$ is the derivative of $q_{r}^{}$ with respect to r, $J_{h}$ is the joule heating term, and $\frac{D}{Dt}$ is the material derivative. 

The relevant boundary conditions were:

(5) $$\begin{array}{l} w=U_{w},\;\Theta =\Theta_{w}\;at\;r=R_{w},\\ w=0,\Theta =\Theta_{d}\;at\;r=R_{d} \end{array}\}.$$

The extra stress tensor *S* was defined as:

(6) $$S=-pI+\mu A_{1}+\alpha_{1}A_{2}+\alpha_{2}A_{1}^{2}+\beta_{1}A_{3}+\beta_{2}\left(A_{1}A_{2}+A_{2}A_{1}\right)+\beta_{3}\left(trA_{1}^{2}\right)A_{1}.$$

where p is the pressure, I is the identity tensor, and μ=μ(Θ) is the coefficient of viscosity $\left(kgm^{-1}s^{-1}\right)$. Here *α*_1_ and *α*_2_ are the second order material constants $\left(kgm^{-1}\right)$, the symbols $\beta_{1},\;\beta_{2}$, and $\beta_{3}$ are the third order material constants $\left(kgm^{-1}s^{-1}\right)$, and tr is the trace operator. The quantities $A_{i}\left(i=1,2,3\right)$ are the Rivlin-Ericksen tensors, which were defined by the recursive relation as follows:

(7) $$A_{0}=I,\;A_{1}=L^{T}+L\;and\;A_{n}=A_{n-1}L^{T}+LA_{n-1}+\frac{DA_{n-1}}{Dt},n=2,3,$$

where T denotes the transpose of the matrix and L=gradV.

Because of interaction of the conducting fluid with the magnetic field, a body force of retarding nature, i.e., J×B was attained. This drag force acting along the z -axis was given by:

(8) $$J\times B=\left(0,0,-\sigma B_{0}^{2}w\right),$$

where $B_{0}$ is the uniform magnetic field applied along the positive radial direction.

Considering Equation (1), Equation (2) was satisfied indicating that the fluid flow is possible. The non-zero components of the extra tensor S are:

(9) $$S_{rr}=\left(2\alpha_{1}+\alpha_{2}\right){\left(\frac{dw}{dr}\right)}^{2},S_{zz}=\alpha_{2}{\left(\frac{dw}{dr}\right)}^{2},S_{rz}=S_{zr}=\left(\beta_{2}+\beta_{3}\right){\left(\frac{dw}{dr}\right)}^{3}+\mu \Theta_{r}\left(\frac{dw}{dr}\right).S_{rr}=\left(2\alpha_{1}+\alpha_{2}\right){\left(\frac{dw}{dr}\right)}^{2},S_{zz}=\alpha_{2}{\left(\frac{dw}{dr}\right)}^{2},S_{rz}=S_{zr}=\left(\beta_{2}+\beta_{3}\right){\left(\frac{dw}{dr}\right)}^{3}+\mu \Theta_{r}\left(\frac{dw}{dr}\right).$$

Making substitution of Equations (8) and (9), the equation of the balance of momentum (Equation (3)) becomes:

(10) $$\frac{-\partial p}{\partial r}=\frac{1}{r}\frac{d}{dr}\left[\left(2\alpha_{1}+\alpha_{2}\right)r{\left(\frac{dw}{dr}\right)}^{2}\right],$$

(11) $$\frac{\partial p}{\partial \theta }=0,$$

(12) $$\frac{\partial p}{\partial z}=\frac{1}{r}\frac{d}{dr}\left(r\mu \left(\theta \right)\frac{dw}{dr}\right)+\frac{1}{r}\frac{d}{dr}\left(2\left(\beta_{2}+\beta_{3}\right)r{\left(\frac{dw}{dr}\right)}^{3}\right)-\sigma B_{0}^{2}w,$$

Equation (12) describes the flow due to the pressure gradient. As the drag of the wire prevails outside the die, the pressure gradient is assumed to be zero i.e., $\frac{\partial p}{\partial z}=0$. So Equation (12) takes the form:

(13) $$2\beta_{0}\frac{1}{r}\frac{d}{dr}\left[r{\left(\frac{dw}{dr}\right)}^{3}\right]+\frac{1}{r}\frac{d}{dr}\left(r\mu \left(\Theta \right)\left(\frac{dw}{dr}\right)\right)-\sigma B_{0}^{2}w=0,$$

where $\beta_{0}=\beta_{1}+\beta_{2}.$

The viscous dissipation term was:

(14) $$\phi =S:\;\nabla V=\mu \left(\Theta \right){\left(\frac{dw}{dr}\right)}^{2}+2\beta_{0}{\left(\frac{dw}{dr}\right)}^{4}.$$

Using the Rosseland approximation for thermal radiation [42] the radiative heat flux was modeled as:

(15) $$q_{r}=-\frac{4\sigma_{1}}{3k_{1}}\frac{d\Theta^{4}}{dr}.$$

Following Pantokratoras and Fang [43], Equation (15) can be written as:

(16) $$q_{r}=-\frac{16\sigma_{1}}{3k_{1}}\Theta^{3}\frac{d\Theta }{dr}.$$

Using Equations (14)–(16), the energy equation (Equation (4)) reads:

(17) $$k\left[\frac{d^{2}\Theta }{dr^{2}}+\frac{1}{r}\frac{d\Theta }{dr}\right]+\mu \left(\Theta \right){\left[\frac{dw}{dr}\right]}^{2}+2\beta_{{}_{0}}{\left[\frac{dw}{dr}\right]}^{4}+\frac{d}{dr}\left[\frac{16\sigma_{1}}{3k_{1}}\Theta^{3}\frac{d\Theta }{dr}\right]+\sigma B_{0}^{2}w^{2}=0.$$

Let us introduce the dimensionless parameters as:

(18) $$\bar{r}=\frac{r}{R_{w}},\bar{w}=\frac{w}{U_{w}},\bar{\Theta }=\frac{\Theta -\Theta_{w}}{\Theta_{d}-\Theta_{w}},M^{2}=\frac{\sigma B_{0}^{2}R_{w}^{2}}{\mu_{0}},\;\bar{\beta_{0}}=\frac{u_{w}^{2}\beta_{0}}{\mu_{0}R_{w}^{2}},\;B_{R}=\frac{\mu_{0}u_{w}^{2}}{k\Theta_{w}},\;\delta =\frac{R_{d}}{R_{w}},\;R=\frac{16\sigma_{1}\Theta_{w}^{3}}{3kk_{1}}$$

## 3. Temperature-Dependent Viscosity

For the temperature-dependent viscosity we used Reynolds’ and Vogel’s model.

### 3.1. Reynolds’ Model 

This is a model that accounts for the temperature-dependent viscosity. For this model, the expression for the temperature dependent viscosity was:

(19) $$\mu \left(\Theta \right)=\mu_{0}e^{-\beta_{0}m\Theta },$$

where *m* is a non-dimensional viscosity parameter associated with the Reynolds’ model.

Using the Equations (18) and (19) in the Equations (13) and (17), and dropping the bar for simplicity, we get the non-dimensional momentum, and energy equations along with the reduced boundary conditions as:

(20) $$e^{-\beta_{0}m\Theta }\left(r\frac{d^{2}w}{dr^{2}}+\frac{dw}{dr}-mr\beta_{0}\frac{d\Theta }{dr}\frac{dw}{dr}\right)+2\beta_{0}\left[3r{\left(\frac{dw}{dr}\right)}^{2}\left(\frac{d^{2}w}{dr^{2}}\right)+{\left(\frac{dw}{dr}\right)}^{3}\right]-M^{2}wr=0,$$

(21) w(1)=0 and Θ(δ)=0,

(22) $$\begin{array}{l} \left(\Theta_{R}-1\right)\left(r\frac{d^{2}\Theta }{dr^{2}}+\frac{d\Theta }{dr}\right)+rB_{r}e^{-\beta_{0}m\Theta }{\left(\frac{dw}{dr}\right)}^{2}+2rB_{r}\beta_{0}{\left(\frac{dw}{dr}\right)}^{4}\\ +rB_{r}\left(\theta_{R}-1\right)\frac{d}{dr}\left\{{\left[1+\Theta \left(\Theta_{R}-1\right)\right]}^{3}\frac{d\Theta }{dr}\right\}+M^{2}B_{r}w^{2}=0, \end{array}$$

(23) Θ(1)=0 and Θ(δ)=1.

### 3.2. Vogel’s Model 

In this model, the expression for the temperature-dependent viscosity was:

(24) $$\mu \left(\Theta \right)=\mu_{1}e^{-\Theta_{w}}e^{\frac{D}{B_{1}+\Theta }}=\mu_{0}e^{\frac{D}{B_{1}+\Theta }},$$

where D and $B_{1}$ are the viscosity parameters affiliated with the Vogel’s model and $\mu_{0}=\mu_{1}e^{-\Theta_{w}}.$ Here it is remarkable to note that the previous authors had considered the first order approximation of the Taylor’s series expansion in Equations (19) and (24). However, we have considered the higher order approximations in Equations (19) and (24) so as to accomplish the characteristic behavior of higher order terms involving the parameters $\beta_{0},m,\Theta ,D,Bi,\Theta_{w}.$ Using the Equations (18) and (24) in the Equations (13) and (17) and dropping the bar for simplicity we get the non-dimensional momentum, and energy equations along with the reduced boundary conditions as:

(25) $$e^{\frac{D}{B_{1}+\Theta }}\left(r\frac{d^{2}w}{dr^{2}}+\frac{dw}{dr}-r\left[\frac{D}{{\left(B_{1}+\Theta \right)}^{2}}\right]\frac{d\Theta }{dr}\frac{dw}{dr}\right)+2\beta_{0}\left[3r{\left(\frac{dw}{dr}\right)}^{2}\left(\frac{d^{2}w}{dr^{2}}\right)+{\left(\frac{dw}{dr}\right)}^{3}\right]-M^{2}wr=0,$$

(26) w(1)=1 and w(δ)=0,

(27) $$\begin{array}{l} \left(\Theta_{R}-1\right)\left(r\frac{d^{2}\Theta }{dr^{2}}+\frac{d\Theta }{dr}\right)+rB_{r}e^{\frac{D}{B_{1}+\Theta }}{\left(\frac{dw}{dr}\right)}^{2}+2rB_{r}\beta_{0}{\left(\frac{dw}{dr}\right)}^{4}+\\ rR\left(\Theta_{R}-1\right)\frac{d}{dr}\left\{{\left[1+\Theta \left(\Theta_{R}-1\right)\right]}^{3}\frac{d\Theta }{dr}\right\}+B_{r}M^{2}w^{2}=0, \end{array}$$

(28) Θ(1)=0 and Θ(δ)=1.

## 4. Convergence of the Method

**Figure 2:**
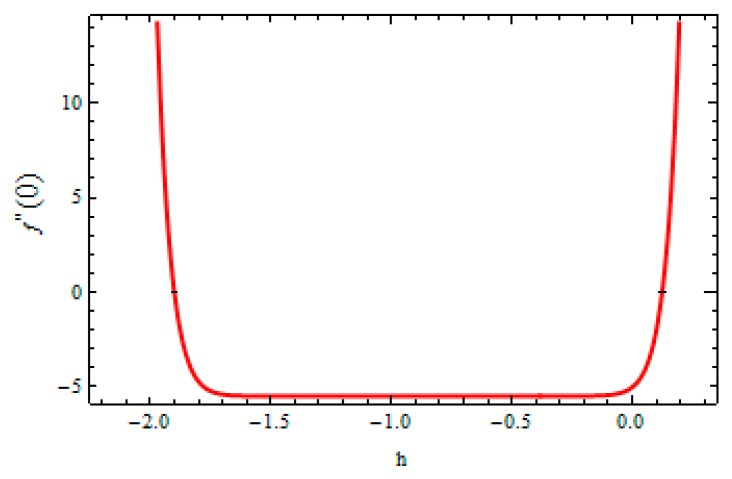
h-curve for velocity field.

**Figure 3:**
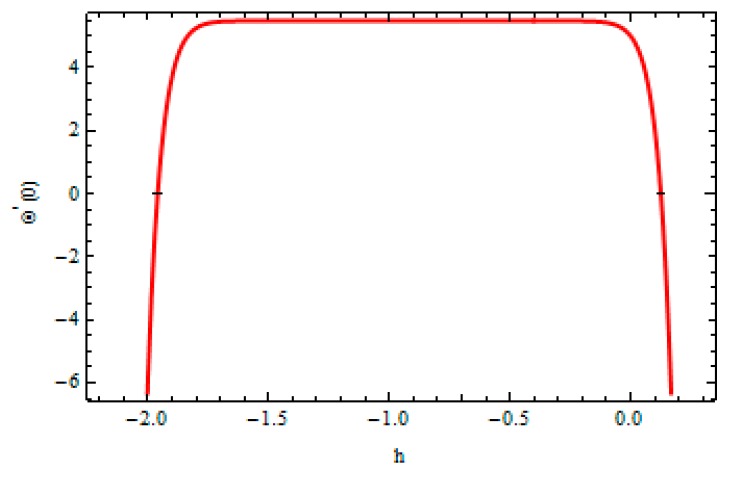
h-curve for temperature field.

In order to validate the method, the convergence of the method is also necessary. For this, the h-curve was drawn to ensure the convergence of the series solution. The calculations were carried out on a personal computer with 4 GB RAM and 2.70 GHz CPU. The code was developed using the computer software MATHEMATICA Zeeshan et at. [44]. To see the range of admissible values of these parameters, the $h_{f}$ and $h_{\theta }$ are plotted in Figure 2 and Figure 3 given by 20^th^ order approximation which took approximately less than a minute in execution. The suitable ranges for $h_{f}$ and $h_{\theta }$ were $-1.5\leq h_{f}\leq -0.3$ and $-1.7\leq h_{\theta }\leq -0.3,$ respectively. 

### Validation of the Method

**Figure 4:**
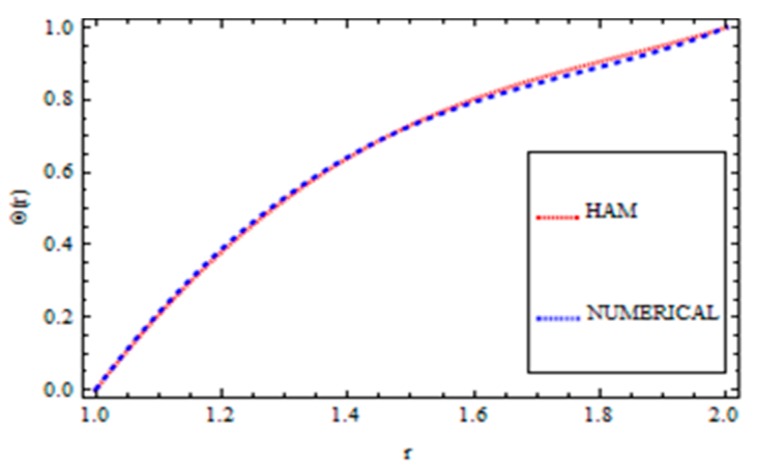
Comparison of the HAM and Numerical Solution for the velocity field (Reynolds’ model).

**Figure 5:**
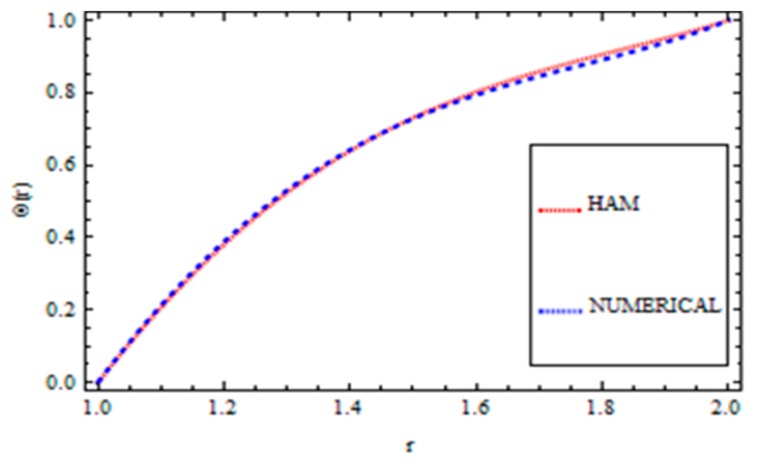
Comparison of the HAM and Numerical Solution for the temperature field (Reynolds’ model).

**Figure 6:**
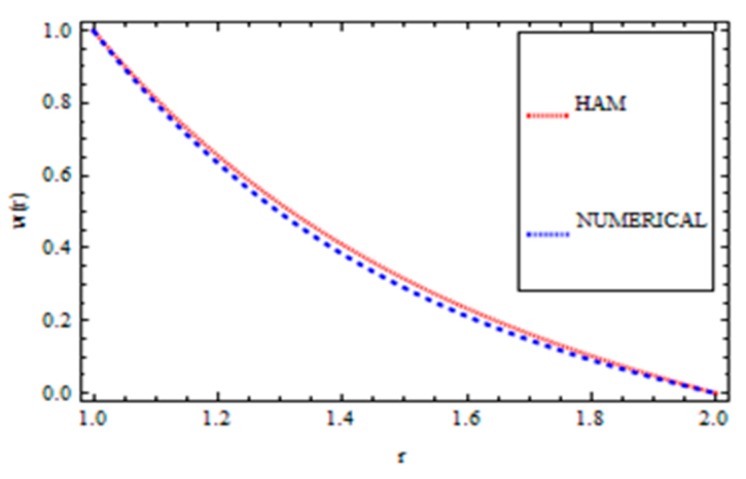
Comparison of the HAM and Numerical Solution for the velocity field (Vogel’s model).

**Figure 7:**
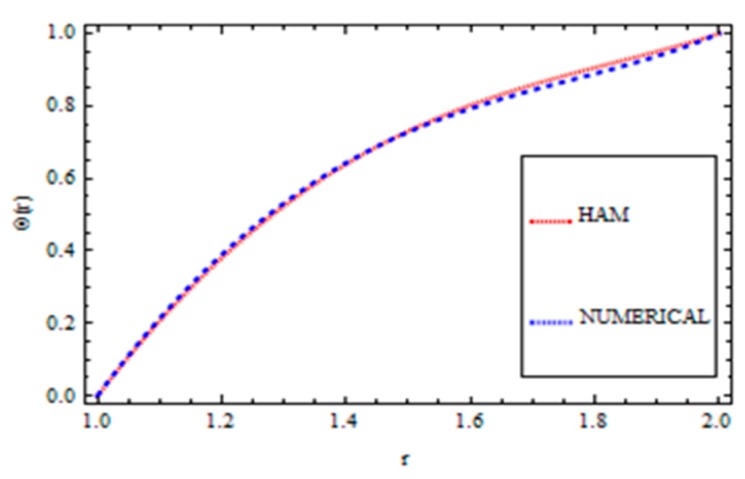
Comparison of the HAM and Numerical Solution for the temperature field (Vogel’s model).

For validation of the results, a numerical method called the *ND*-solve method was applied. From this method, we had good agreement as shown in Figure 4, Figure 5, Figure 6 and Figure 7. In order to ensure the accuracy of our results, the present results were also compared quantitatively with the published work of Zeeshan et al. [44], as shown in Table 1. This comparison confirmed that our analytical results were in excellent agreement for the proposed values of the parameter and therefore we are confident about the accuracy and generality of our results.

## 5. Results and Discussion 

The influence of the radiative linear as well as non-linear heat transfer on the wire coating using a third grade fluid has been investigated with variable viscosities subject to joule heating. For the temperature dependent viscosity, the Reynolds’ model and Vogel’s model has been used. The modified governing boundary layer equations along with the boundary conditions were solved using the Homotopy Analysis Method (HAM). The analytical results revealed the effect of the thermal radiation (linear as well as non-linear) on the velocity, temperature, and heat transfer in the process of wire coating in the presence of a magnetic field and hence discussed in detail. 

### 5.1. Reynolds’ Model

**Figure 8:**
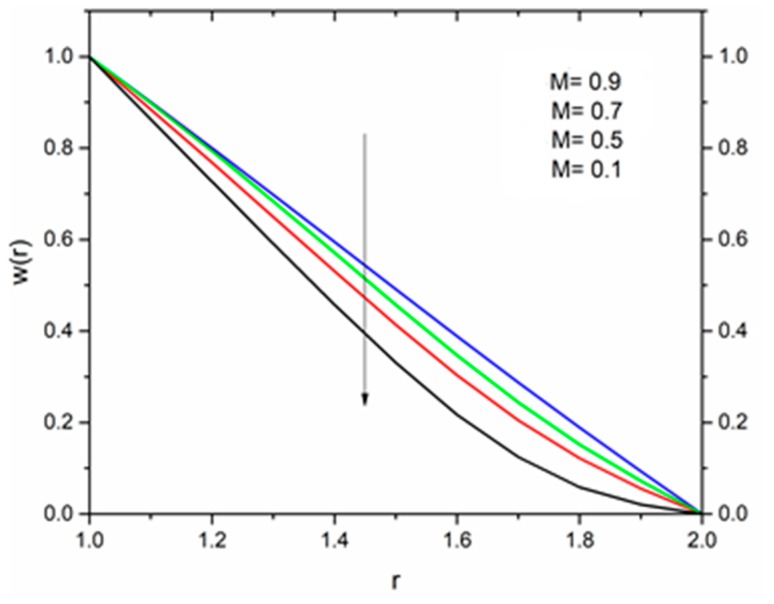
Influence of the magnetic parameter M on the velocity for $\Theta_{R}=1.0,\;\beta_{0}=0.01,B_{r}=10,\;R=1,\;m=5$ (Reynolds’ model).

**Figure 9:**
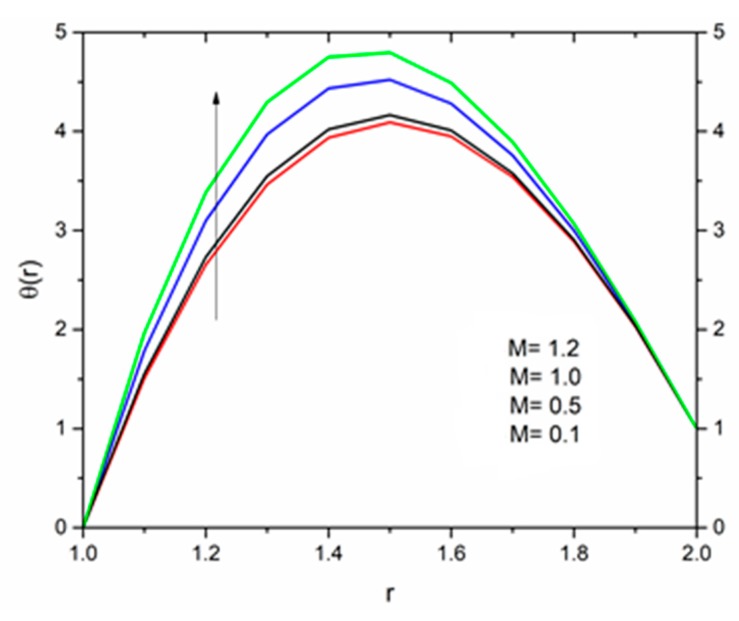
Influence of M on the temperature for $\Theta_{R}=1.0,\;\beta_{0}=0.01,m=5,\;B_{r}=10,\;R=1.0$ (Reynolds’ model).

**Figure 10:**
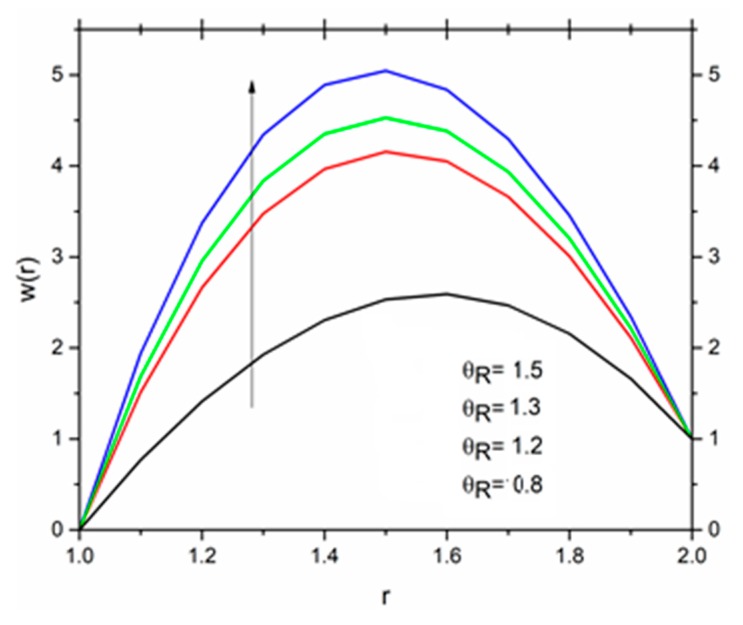
Influence of the temperature ratio parameter $\Theta_{R}$ on the velocity for M = 1.0, β_0_ = 0.01, B_r_ = 10, R = 1, m = 5 (Reynolds’ model).

**Figure 11:**
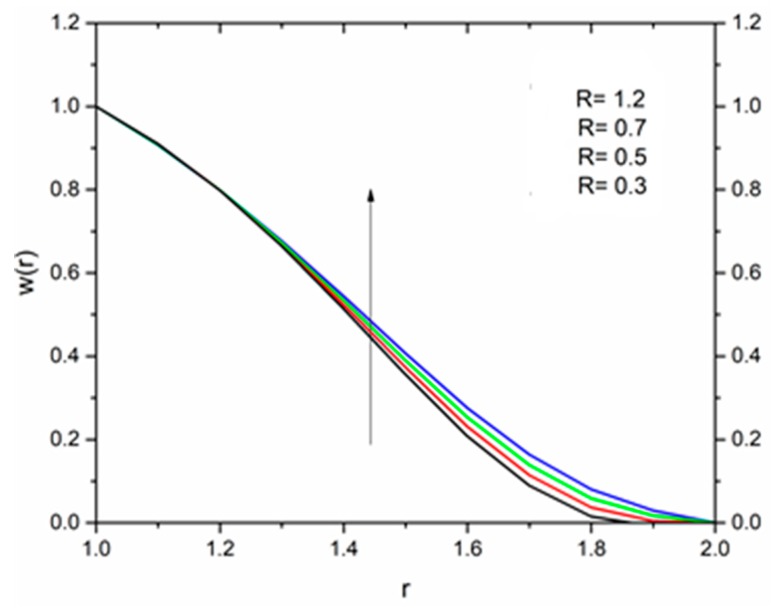
Influence of the radiation parameter R on the velocity for M = 1.0, β_0_ = 0.01, m = 5, B_r_ = 10, Θ_R_ = 1.1 (Reynolds’ model).

The effect of the magnetic parameter M on the velocity and temperature profiles has been shown in Figure 8 and Figure 9 respectively. The velocity profiles decreased as the magnetic parameter increased. This was due to the resistive Lorentz’s force which came into play as a result of the interaction of the magnetic field with the conducting fluid, used as coating material. Figure 9 shows that the magnetic field had an accelerating effect on the temperature distribution with higher temperatures observed near the surface of the wire and thereafter, decreased, showing the shear thickening effect. The variation of fluid velocity for various values of the temperature parameter $\Theta_{R}$ and radiation parameter R is shown in Figure 10 and Figure 11 respectively. It was observed that the velocity of the coating fluid accelerated near the surface of the wire (r≤1.4) when the temperature parameter increased, and a reverse trend was observed towards the die surface as shown in Figure 10. The influence of the thermal radiation parameter *R* on the velocity behavior is depicted in Figure 11. From this figure it is understood that velocity of the polymer within the die increased significantly due to the increasing values of R in the presence of a lower magnetic field and moderated viscous heating. 

**Figure 12:**
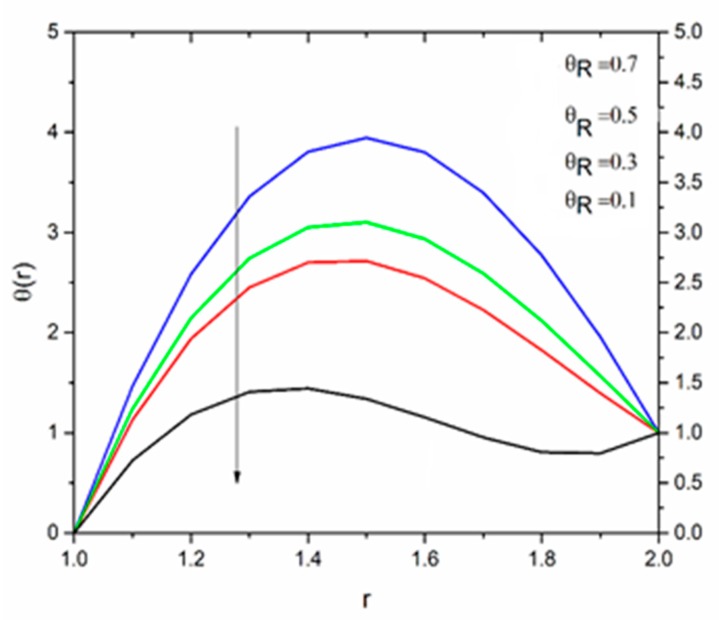
Influence of $\Theta_{R}$ on the temperature for M = 1.0, β_0_ = 0.01, m = 5, B_r_ = 10, R = 1.0 (Reynolds’ model).

**Figure 13:**
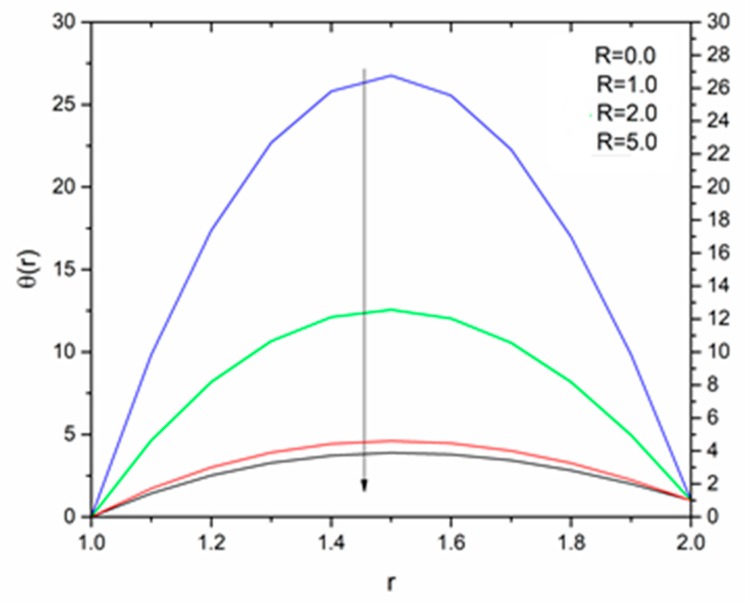
Influence of R on the temperature for M = 1.0, β_0_ = 0.01, m = 5, B_r_ = 10 with Θ_R_ = 1.1 (Reynolds’ model).

**Figure 14:**
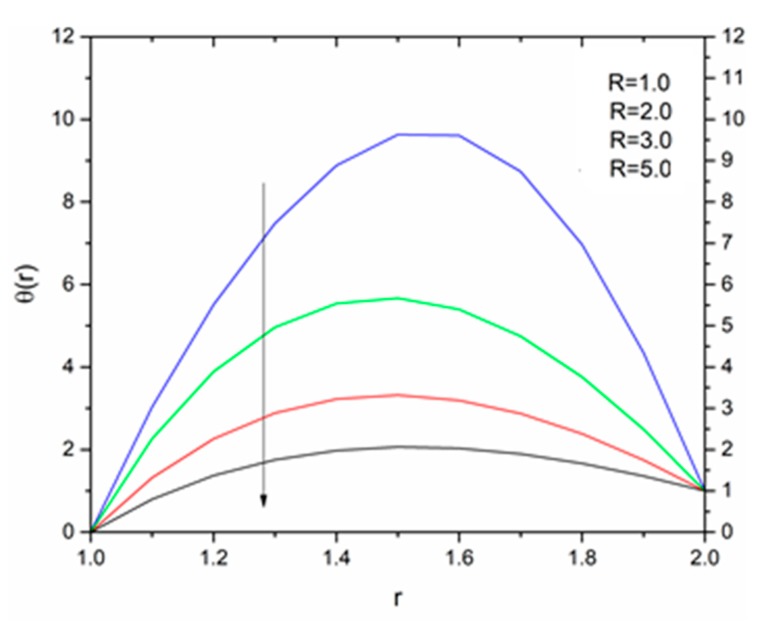
Influence of R on the temperature for M = 1.0, β_0_ = 0.01, m = 5, B_r_ = 10 with Θ_R_ = 1.5 (Reynolds’ model).

**Figure 15:**
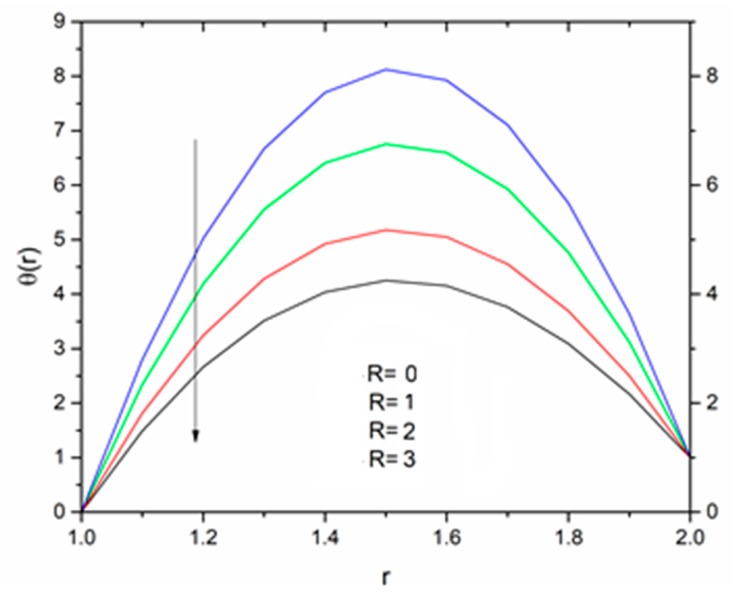
Influence of R on the temperature for M = 1.0, β_0_ = 0.01, m = 5, B_r_ = 10 with Θ_R_ = 2.5 (Reynolds’ model).

Figure 12 is sketched to show the effect of the temperature parameter $\Theta_{R}$ on the fluid temperature. It is interesting to note that the fluid temperature decreased with increasing values of $\Theta_{R}$. The effect of the radiation parameter R on the fluid temperature for different values of the temperature parameter $\Theta_{R}$ is shown in Figure 13, Figure 14 and Figure 15. We can say that the temperature of the coating fluid decreased with increasing R. It was also observed that the decrease in fluid temperature was prominent for $\Theta_{R}$ = 1.2 as compared to $\Theta_{R}$ = 1.6 and $\Theta_{R}$ = 2.5. It was clear that the heat transfer rate was more for $\Theta_{R}$ = 1.2 comparatively to $\Theta_{R}$ = 1.6 and $\Theta_{R}$ = 2.5. 

### 5.2. Vogel’s Model

**Figure 16:**
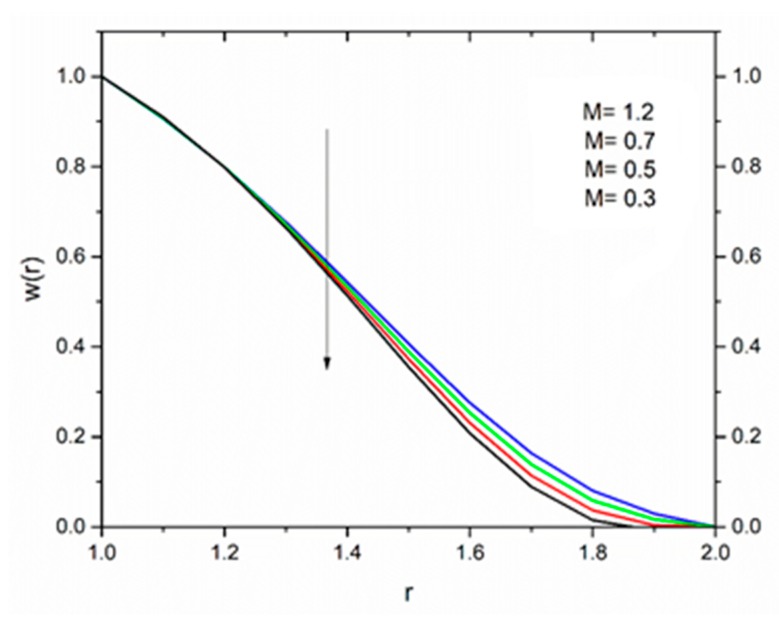
Influence of M on velocity for Θ_R_ = 1.0, β_0_ = 0.01, m = 5, B_r_ = 1.0, R = 1.0, Ω = 1.0 (Vogel’s model).

**Figure 17:**
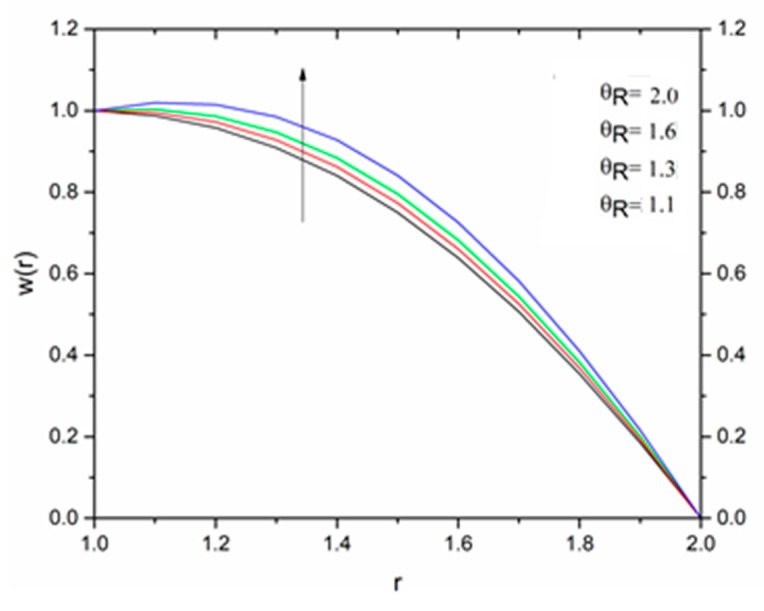
Influence of Θ_R_ on the velocity for M = 1.0, β_0_ = 0.01, m = 5, B_r_ = 1.0, R = 1.0, Ω = 1.0 (Vogel’s model).

**Figure 18:**
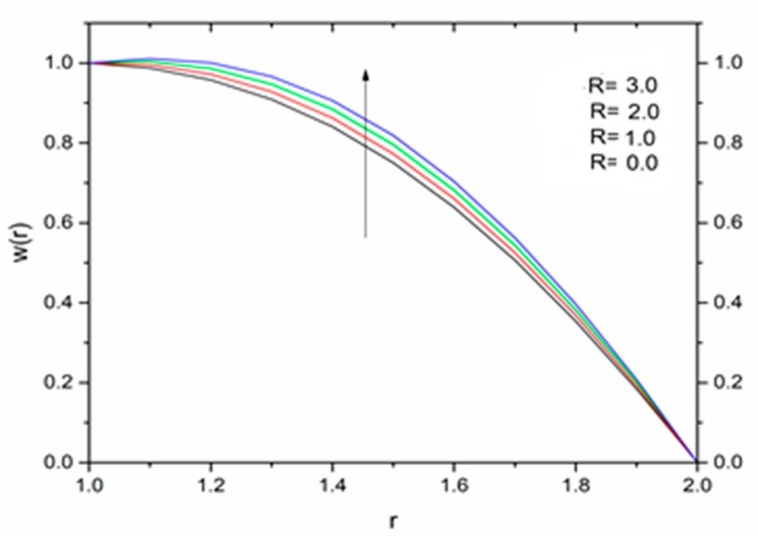
Influence of R on the velocity for M = 1.0, β_0_ = 0.01, B_r_ = 1.0, Θ_R_ = 1.1, Ω = 1.0 (Vogel’s model).

**Figure 19:**
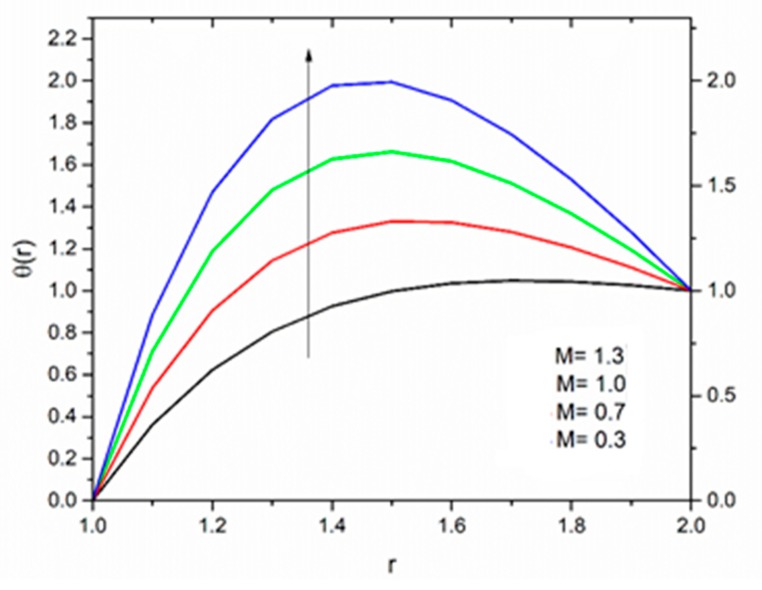
Influence of M on the temperature for Θ_R_ = 1.0, β_0_ = 0.01, B_r_ = 1.0, R = 1.0, Ω = 1.0 (Vogel’s model).

**Figure 20:**
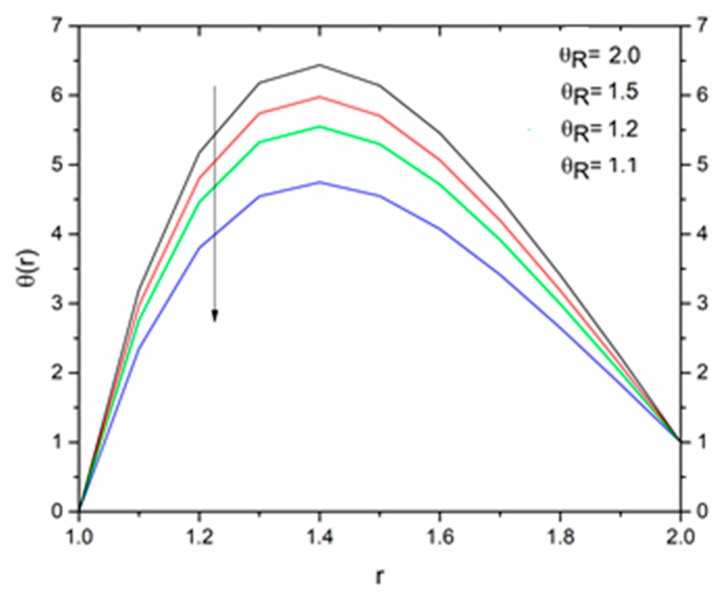
Influence of $\Theta_{R}$ on the temperature for M = 1.0, β_0_ = 0.01, B_r_ = 1.0, R = 1.0, Ω = 1.0 (Vogel’s model).

Figure 16 show the influence of the magnetic parameter M on the velocity. The effect of the magnetic field was the same on the velocity profiles as discussed in Reynolds’ model. It has been discussed that the fluid velocity varied in response to the linear as well as non-linear thermal radiation in the presence of variable viscosity in the Reynolds’ model case. The influence of the radiation parameter as well as the temperature ratio parameter on the coating fluid is shown in Figure 17 and Figure 18 respectively. It has been observed that the velocity of the fluid within the die increased as the radiation parameter R and temperature parameter $\Theta_{R}$ increased. The effect of magnetic parameter on the temperature profile is same as discussed in Reynolds’ model case as shown in Figure 19. The effect of temperature ratio parameter $\Theta_{R}$ on the temperature distribution is displayed in Figure 20. It has been clearly observed that fluid temperature decreased due to an increase in the temperature ratio parameter $\Theta_{R}$.

**Figure 21:**
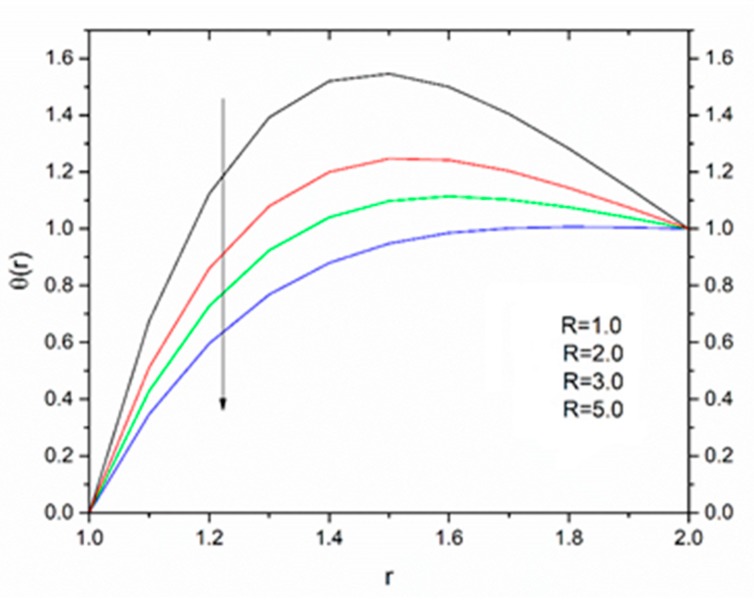
Influence of R on the temperature for M = 1.0, β_0_ = 0.01, B_r_ = 1.0, θ_R_ = 1.1, Ω = 1.0 (Vogel’s model).

**Figure 22:**
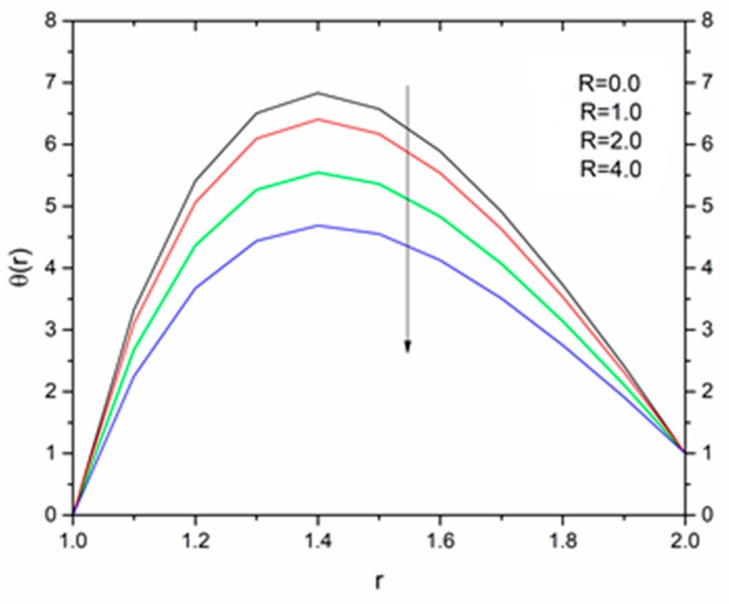
Influence of R on the temperature for M = 1.0, β_0_ = 0.01, B_r_ = 1.0, θ_R_ = 1.5, Ω = 1.0 (Vogel’s model).

**Figure 23:**
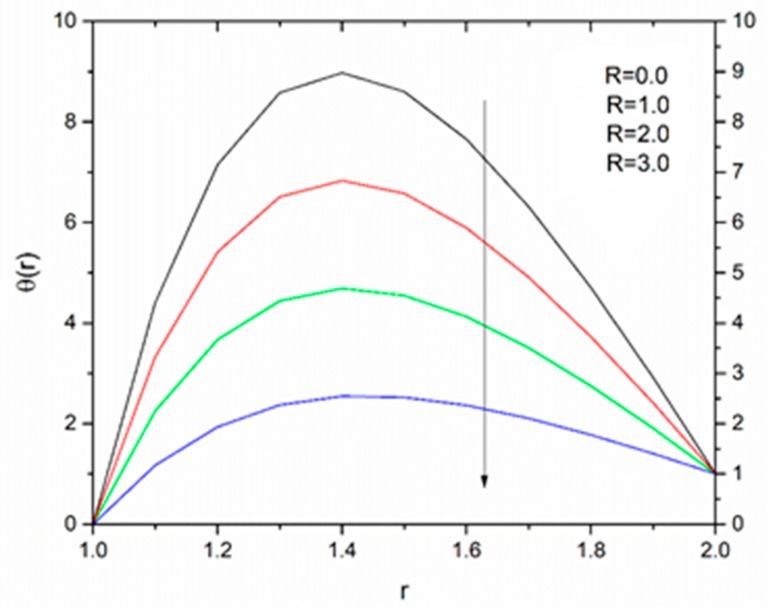
Influence of R on the temperature for M = 1.0, β_0_ = 0.01, B_r_ = 1.0, θ_R_ = 2.5, Ω = 1.0 (Vogel’s model).

The effect of the radiation parameter R on the temperature distribution is shown in Figure 21, Figure 22 and Figure 23. It was noticed from this observation that the fluid temperature decreased as R increased in response to Vogel’s viscosity model. An important point to keep in mind in this regard is that the decreasing trend in fluid temperature was prominent and symmetric at R = 1.4 for $\Theta_{R}$ = 1.5 compared to $\Theta_{R}$ = 1.2 and $\Theta_{R}$ = 2.5 as illustrated in Figure 21, Figure 22 and Figure 23.

## 6. Conclusions

The influence of the linear as well as non-linear thermal radiation along with the pertinent parameter on the coating fluid in wire coating that was associated with joule heating has been discussed with the help of graphs. In the recent study, the non-dimensional momentum and energy equation along with the reduced boundary condition has been solved analytically using the Homotopy Asymptotic Method (HAM). The analytical results achieved in the present study agree quantitatively with the numerical results (*ND*-Solvem method) and previously published results. It has been observed that the velocity of the coating fluid accelerated near the surface of the wire with an increase in $\Theta_{R}$. It is interesting to note that the velocity of the coating fluid increased with an increase in *R* in the presence of a lower magnetic field and moderate viscous heating. The fluid temperature decreased with increasing values of R and $\Theta_{R}$, in both the Reynolds’ model and Vogel’s model cases.

## Figures and Tables

**Table 1:** Comparison of the present work with the published work of Zeeshan et al. [43]. Θ_*R*_ = 1.0, β_0_ = 0.01, B_r_ = 10, R = 1, m = 5.

S. No	Published Work	Present Work
1	1	1
1.1	0.906702201	0.906702202
1.2	0.798963328	0.798963327
1.3	0.676887100	0.676887101
1.4	0.543737426	0.543774255
1.5	0.406571921	0.4065719210
1.6	0.275849318	0.275849317
1.7	0.163688021	0.1636880211
1.8	0.080480501	0.080480502
1.9	0.0296124455	0.0296124456
2.0	1.23245E-26	0.2138E-30

## Nomenclature

$R_{w}$	Wire radius (m)	M	Magnetic parameter	(r,z)	Co-ordinates system
U_w_	Dragging velocity (ms^−1^)	$\theta_{w}$	Wire temperature (K)	L	Length of the die (m)
$\theta_{R}$	Temperature parameter	$\sigma_{1}$	Stefan-Boltzman constant (Wm^−2^K^−4^)	$\mu_{0}$	Reference viscosity (N sm^−2^)
R	Radiation parameter	$\frac{\mathrm{dp}}{\mathrm{dz}}$	Constant pressure gradient	$B_{0}$	Uniform magnetic field
$R_{d}$	Radius of the die (m)	$\beta_{0}$	Non-Newtonian Parameter	ρ	Density of the fluid
$q_{r}$	Radiative heat flux (Wm^−2^)	ϕ	Dissipation function (Wm^−2^)	w	Velocity of the fluid (ms^−1^)
θ	Fluid temperature (K)	$\alpha_{1},\alpha_{2},\beta_{1},\beta_{2}$	Material constants	$\frac{D}{\mathrm{Dt}}$	Substantial derivative
p	Pressure	$B_{r}$	Brinkmen number	$J_{h}$	Joule heating
$\Theta_{d}$	Die temperature (k)	m	Reynolds’ model viscosity parameter	F	Viscous force per unit volume (Nm^−3^)
$\theta_{d}$	Die temperature (K)	k	Thermal conductivity	$C_{p}$	Specific heat at constant pressure
δ	Wire coating aspect ratio	$k_{1}$	Mean absorption coefficient (m^-1^)	μ	Dynamic viscosity (N sm^−2^)
